# Commentary: “Multimodal Theories of Recognition and Their Relation to Molyneux's Question”

**DOI:** 10.3389/fpsyg.2015.01792

**Published:** 2015-11-19

**Authors:** John Schwenkler

**Affiliations:** Department of Philosophy, Florida State UniversityTallahassee, FL, USA

**Keywords:** Molyneux, multisensory perception, nativism debate, empiricism, associative learning, intersensory integration, cross-modal perception

Altieri (2015) discusses the relevance of experimental work on cross-modal recognition to a question raised by the Irish politician William Molyneux and discussed in John Locke's *Essay Concerning Human Understanding*:

“Suppose a man born blind, and now adult, and taught by his touch to distinguish between a Cube, and a Sphere …, so as to tell, when he felt one and t'other, which is the Cube, which the Sphere. Suppose then the Cube and Sphere placed on a Table, and the Blind Man to be made to see. *Qu6re*, whether by his sight, before he touched them, he could now distinguish, and tell, which is the Globe, which the Cube.”

Locke (1694/1979).

Altieri argues persuasively that answers to Molyneux's question do not break down neatly across nativist vs. empiricist lines, as only when a nativist view of concepts is combined with the further thesis that sensory representations are either innately cross-modal or automatically translated into a cross-modally common code, does such a view predict an affirmative answer to Molyneux's question. Absent such an assumption, the nativist can predict a negative answer instead.

Altieri is also correct in endorsing criticisms I have raised (Schwenkler, 2012, 2013; Connolly, 2013) against the resolution of Molyneux's question attempted by Held et al. (2011). In this study, newly sighted individuals could match seen objects with seen objects and felt with felt, but could not match seen objects with felt ones, leading the authors to conclude that “the answer to Molyneux's question is likely negative,” as any innate link between vision and touch “is insufficient for reconciling the identity of the separate sensory representations” (Held et al., 2011). But given the evidence that newly sighted patients have only a limited capacity to form 3D visual representations of complex objects (Fine et al., 2003; Ostrovsky et al., 2009), these individuals' failure in the cross-modal matching task could have been due to a purely visual deficit. Therefore, the study does not establish anything about the relationship between visual and tactile representations.

Less persuasive, however, is Altieri's proposal to substitute for Molyneux's question a test of whether newly sighted individuals exhibit the McGurk effect (McGurk and MacDonald, 1976). Altieri suggests that the latter effect is evidence of a cross-modal connection between the auditory and visual modalities, and so if it is observed prior to any relevant perceptual learning, the association that underlies it must be innate. But not just any theory that predicts an affirmative answer to Molyneux's original question will also predict that newly sighted individuals will exhibit the McGurk effect. This is because there could be differences in how phonemes and shapes, respectively, are cross-modally coded, or in the nature of the cross-modal connections between vision and hearing as opposed to those between vision and touch. This makes Altieri's question different from Molyneux's not just in the paradigms they employ, but also in which hypotheses they manage to test.

More precisely, the difficulty for Altieri's proposal is that there can be different versions of what he calls the *Common Code Theory*, i.e., the theory holding that perceptual representations are both innate and amodal or cross-modal. First, versions of the Common Code Theory may differ in which perceptible properties they take to be commonly coded: e.g., it might be thought that low-level spatial and temporal properties are commonly coded, whereas high-level ones like abstract category membership are not. Second, versions of the theory may differ in which sensory modalities they take this code to be shared between: e.g., it might be that vision and touch share an innately common code, whereas the connections between these modalities and those of smell and taste have to be learned (It is an interesting question, which I can't explore here, how exactly the Gibsonian theories of “direct perception” that Altieri discusses would fit into this framework). Importantly, the only version of the Common Coding Theory that must predict an affirmative answer to Molyneux's original question is a version that postulates an unlearned amodal or cross-modal representation of *spatial* properties that is common to *sight and touch*. Such a modest thesis would not entail that there is also such a connection between the representation of phonemic properties in sight and hearing, and so would not require making any prediction at all regarding the McGurk effect in newly sighted individuals.

For it to work, then, Altieri's proposal needs to be modified so that the stimuli are more appropriate to the hypothesis at stake in Molyneux's original question. One way to do this would be to explore sensory dominance effects in the interaction between vision and touch. The McGurk effect is just one instance of a wide range of intersensory effects discovered in recent decades, and some of these concern cross-modal interactions within the perception of spatial properties rather than phonemic ones (e.g., the ventriloquist effect; Alais and Burr, 2004), and interactions between touch and vision rather than vision and hearing (e.g., the rubber hand illusion; Pavani et al., 2000)^1^. A version of the Common Coding Theory that predicts an affirmative answer to Molyneux's question might also predict that phenomena like these would be observed in the newly sighted, as prior to any learned associations between vision and other modalities their sensory systems would still resolve competition between the commonly coded information present in these modalities. It is possible, however, that newly sighted individuals might show different patterns of sensory dominance than experienced seers, e.g., visual experience might more often be captured by competing tactile stimuli (Violentyev et al., 2005). Still, Altieri is right that testing for some such cross-modal interactions would be a promising way forward on this question.

## Author contributions

JS is the sole author of this paper.

## Acknowledgments

Thanks to Matthew Fulkerson and Joshua Shepherd for reading a draft of the MS, and to a referee for valuable feedback.

### Conflict of interest statement

The author declares that the research was conducted in the absence of any commercial or financial relationships that could be construed as a potential conflict of interest.

## Footnotes

^1^On intersensory effects between vision and taste, see Spence et al., 2010; and cf. Gagnon et al., 2015, for related work involving the congenitally blind.
